# Multimorbidity from Chronic Conditions among Adults in Urban Slums: The AWI-Gen Nairobi Site Study Findings

**DOI:** 10.5334/gh.771

**Published:** 2021-01-20

**Authors:** Shukri F. Mohamed, Tilahun Nigatu Haregu, Olalekan A. Uthman, Christopher Khayeka-Wandabwa, Stella Kagwiria Muthuri, Gershim Asiki, Catherine Kyobutungi, Paramjit Gill

**Affiliations:** 1 African Population and Health Research Center (APHRC), Nairobi, KE; 2 Academic Unit of Primary Care (AUPC) and the NIHR Global Health Research Unit on Improving Health in Slums, University of Warwick, Coventry, UK; 3 Non-Communicable Disease Unit, School of Population and Global Health, University of Melbourne, AU; 4 Warwick-Centre for Applied Health Research and Delivery (WCAHRD), Warwick Medical School, University of Warwick, Coventry, UK; 5 School of Pharmaceutical Science and Technology (SPST), Health Sciences Platform, Tianjin University, Tianjin, CN

**Keywords:** multimorbidity, chronic conditions, urban slum, anthropometry, ethnicity

## Abstract

**Background:**

In the era of double burden of infectious and non-communicable diseases in sub-Saharan Africa, the burden of multimorbidity is likely to be common. However, there is limited evidence on the burden and its associated factors in the sub-Saharan African context.

**Objective:**

The aim of this study was to determine the levels and identify determinants of multimorbidity from chronic conditions in two urban slums in Nairobi.

**Methods:**

Data collected from 2003 study participants aged 40–60 years in two urban slums of the Nairobi Urban Health and Demographic Surveillance System in 2015 were used. Using self-report, anthropometry and key biomarkers, data on 16 conditions including chronic diseases, behavioral disorders and metabolic abnormalities were gathered. Lifetime multimorbidity defined by the occurrence of at least two chronic conditions in an individual at any time during their life course was computed. Factors associated with lifetime multimorbidity were identified using multiple logistic regression.

**Findings:**

A total of 2,081 chronic conditions were identified among 1,302 individuals. While 701 (35.0%) had no chronic condition, single morbidity was reported in 726 (36.2%) of the study population. The overall prevalence of lifetime multimorbidity was 28.7%. The prevalence of dyads and triads of simultaneous occurrences of conditions (episodic multimorbidity) was 20.8% and 6.1%, respectively. Single morbidity was positively associated with gender and alcohol consumption; and negatively associated with employment. Women, older people, the unemployed, current smokers and current alcohol consumers had higher levels of lifetime multimorbidity in the study population.

**Interpretation:**

The findings of this study indicate that a considerable proportion of adults living in urban slums experience multimorbidity from chronic conditions. Further studies with a better rigor to establish temporal associations between socio-demographic factors and the occurrence of chronic conditions are needed to explore the impacts and implications on health status and health system.

## Background

In the era of double burden of infectious and non-communicable diseases (NCDs), simultaneous occurrence of two or more diseases (multimorbidity) is bound to happen in an individual. This is especially true in low and middle-income countries (LMICs), which still bear the bulk of the burden for infectious disease while also battling a new threat from the rapid emergence of non-communicable diseases [1,2,3].

Multimorbidity, may occur simultaneously at the same time (episodic multimorbidity) or during their lifetime (lifetime multimorbidity) and is reported to be more common among older adults [4,5]. A study examining the age and socioeconomic distribution of multimorbidity in LMICs showed that the prevalence of multimorbidity is fairly high in LMICs (7.8%) [5]. Multimorbidity increases costs in the healthcare systems and to patients alongside gaps in quality of healthcare since it typically challenges the single-disease framework, which most healthcare delivery systems adopt, and how health research and interventions are accustomed to [6]. It is also associated with increased risk of mortality, disability, poor functional status, poor quality of life, and adverse drug events often occasioned by polypharmacy [7,8].

Multimorbidity research has received increasing attention in recent years but more so in the developed country settings and among older adults aged 60 years and above. With the increasing prevalence of NCDs occurring among younger age groups (40–60 years), especially in LMICs, the occurrence of multimorbidity is expected to rise. Population-based estimates among the younger populations (≤60 years) in sub-Saharan Africa is lacking. Only a few studies have highlighted this problem in some settings in Africa [9,10,11]. Almost one billion people are estimated to live in slum households and people in slums have worse health than those in non-slum areas [12]. Slum health is a neglected research area and the need for further research has been highlighted. In light of this, the objective of this study was to determine the magnitude of and identify determinants of lifetime multimorbidity from chronic conditions among adults 40–60 years of age in an urban slum setting.

## Methods

### Study design and setting

This study draws on data collected from 2003 study participants between the ages of 40 and 60 years in the Nairobi Urban Health & Demographic Surveillance System (NUHDSS), a pioneer urban-based Health and Demographic Surveillance System in sub-Saharan Africa (SSA) [13]. Through a population based-cross-sectional survey sociodemographic, anthropometric, biomedical and genetic data were collected among 10,702 adults across six Health and Demographic Surveillance System in five sub-Saharan Africa countries (Kenya, Ghana, Burkina Faso and South Africa) in the Africa Wits-INDEPTH partnership for Genomic Studies (AWI-Gen) [14]. The study included approximately 2,000 adults aged 40–60 years per site with nearly equal proportions of both males and females, selected randomly from updated census databases maintained by each site [15]. The broader aim of the study was to identify the contribution of environmental and genetic factors to body composition, including obesity, and susceptibility to cardio-metabolic disease [16,17,18].

### Data collection, measurements, and definition of variables

Data on chronic conditions was collected using self-report and direct measures of anthropometry and key biomarkers. Trained field workers at centralized clinical centers located in the study communities administered an extensive validated questionnaire to each participant. The questionnaire had three main sections: Demography, Health History and Anthropometry, and Sample Collection (blood and urine). Data on weight, height, waist circumference and hip circumference were collected. Waist circumference was measured using SECA 201™ circumference measurement tape. Weight and height were measured using a validated SECA 874™ weighing machine and a portable stadiometer (Seca 213™), respectively. Screening blood pressure measurements were taken using a validated Omron™ M10-IT blood pressure machine. An appropriately sized cuff was placed on the non-dominant arm after the subject had been seated for at least five minutes. Three BP measurements were taken over a five minute period and the mean of the last two measurements was recorded as the screening BP value. It is important to note, only variables and other related parameters relevant to the presented study objective were considered.

### Chronic conditions

In this analysis, we have included 16 conditions encompassing chronic disease, behavioral disorders and metabolic abnormalities. Most of these were self-reported – having asked the participant if they had ever been told by a health worker in the Kenyan context that they had that particular chronic condition [19,20]. Others, such as hypertension and obesity, were based on direct measurements of blood pressure and body mass index. Alcohol disorder was defined as when a participant agreed with either one of the following three statement: feeling that they needed to cut down on their drinking, being annoyed by criticism to their drinking, or having an alcoholic drink first thing in the morning. The assessment method used, and the respective questions asked are shown in Table 1.

**Table 1:** Chronic diseases and their measurement in the study.

S#	Disease condition	Measurement

**1**	Tuberculosis	Self-report: Ever been told that you have TB?
**2**	HIV infection	Self-report & test: Tested HIV+.
**3**	Diabetes	Self-report: Ever been told that you have diabetes?
**4**	Stroke	Self-report: Ever been told that you had a stroke?
**5**	Hypertension	Self-reported & measured: measured SBP/DBP>140/90 or on treatment.
**6**	Angina	Self-report: Ever been told that you had angina?
**7**	Heart attack	Self-report: Ever been told that you had a heart attack?
**8**	Congestive heart failure (CHF)	Self-report: Ever been told that you had CHF?
**9**	High cholesterol	Self-report: Ever been told you have high cholesterol?
**10**	Thyroid disease	Self-report: Ever been told you have thyroid disease?
**11**	Kidney disease	Self-report: Ever been told you have kidney disease?
**12**	Cancer	Self-report: Ever been told you have breast, cervical, prostate or other cancers?
**13**	Asthma/reactive air disease	Self-report: Have had asthma or reactive air disease?
**14**	Alcohol disorder	Self-report: Ever felt you had to cut down on your drinking/people annoyed you by criticising your drinking/bad or guilty about your drinking/you needed an alcohol drink first thing in the morning to steady your nerves or get rid of a hangover?
**15**	Drug use	Self-report: Ever taken marijuana, methamphetamines, cocaine or any other drugs?
**16**	Obesity	Measured: By BMI ≥ 30

SBP = Systolic blood pressure, DBP = Diastolic blood pressure.

### Definition of multimorbidity

In this study, multimorbidity was considered as the co-occurrence of two or more of the 16 chronic condition in an individual [21]. Lifetime multimorbidity was defined by the occurrence of at least two chronic conditions in an individual over their lifetime, that is, the proportion of adults 40–60 years that have ever had two or more of the 16 chronic conditions at some time in their life.

### Data analysis

Descriptive statistics were used to determine the prevalence of individual chronic conditions and number of chronic conditions per subject, for the study population, as well as the prevalence of multimorbidity. We computed the number of study subjects who had none, one, two, three, and four or more conditions as a proportion of the total number of study subjects included.

The prevalence of multimorbidity was then related to the basic sociodemographic characteristics including sex (males and females) and age in five-year categories (40–45, 46–50, 51–55, and 56–60). Lifetime prevalence was assessed for individual chronic conditions as well as combinations of the conditions. Multinomial logistic regression models were fitted to examine the association between lifetime multimorbidity and basic sociodemographic characteristics. Variables used in the final model included sex, age, education level, ethnicity, employment status, wealth, current use of alcohol, current smoking, unhealthy diet and work that involves sitting.

## Results

### Background characteristics of the study population

This study included 2003 healthy adults between 40 and 60 years of age, with slightly more women (54.0%). The mean (SD) age of the participants was 48.8 years and 57.5% had attained a primary level of education. Approximately half (47.3%) were self-employed and about one-third (31.2%) were engaged in informal employment. More details on demographic and socioeconomic characteristics are reported elsewhere [13].

### Prevalence of individual chronic conditions

A total of 2,081 episodes of chronic conditions were identified in the study population. The most prevalent ones were hypertension (22.9%) and obesity (19.9%). Drug use (12.8%), HIV (12.1%), tuberculosis (10.9%) and alcohol disorders (10.9%) which affected about one in every nine adults followed these. These first six chronic conditions accounted for 89.5% of the total number of chronic conditions identified. Diabetes, asthma/reactive air disease, angina, and kidney disease were reported in 1.8–3.1% of the study population. Details of the prevalence of the 16 chronic conditions of interest are shown in Table 2.

**Table 2:** Life-time prevalence of the 16 chronic disease conditions.

S#	Disease condition	#	%

**1**	Hypertension	458	22.9
**2**	Obesity	398	19.9
**3**	Drug use	257	12.8
**4**	HIV	243	12.1
**5**	Tuberculosis	219	10.9
**6**	Alcohol disorder	219	10.9
**7**	Diabetes	62	3.1
**8**	Asthma/reactive air disease	63	3.1
**9**	Angina	50	2.6
**10**	Kidney disease	37	1.8
**11**	Stroke	17	0.8
**12**	Cancer	14	0.7
**13**	Thyroid disease	13	0.6
**14**	Heart attack	11	0.5
**15**	Congestive heart failure	11	0.5
**16**	High cholesterol	9	0.4
		2081	

### Prevalence of ever having at least one chronic condition

More than one-third (35.0%) of the study population had never had any one of the 16 chronic conditions included in these analyses. The reported absence of any chronic condition was higher in men (38.9%) than in women (31.6%) (P = 0.001), and higher in those 50 years old or less (36.7%) compared to those greater than 50 years (32.7%) (P = 0.025). Consequently, the prevalence of at least one chronic condition was higher in women and the older age groups. Prevalence of at least one chronic condition is shown in Table 3.

**Table 3:** Prevalence of at least one chronic condition by background characteristics.

	Categories	Have at least one chronic condition?	Total (N)

**Sex**	Men	61.1%	922
	Women	68.4%	1,081
**Age**	40–45 years	63.7%	724
	46–50 years	62.4%	530
	51–55 years	69.0%	436
	56–60 years	66.8%	313
**Education**	No formal education	66.9%	154
	Primary education	67.2%	1,151
	Secondary education	61.3%	672
	Tertiary education	53.8%	26
**Employment**	Self-employed	67.4%	946
	Full-time employed	52.8%	267
	Part-time employed	66.7%	45
	Informal employment	64.2%	623
	Unemployed	76.5%	119
**Wealth status**	Poorest	70.1%	241
	Second	62.1%	451
	Third	64.4%	464
	Fourth	68.6%	405
	Richest	62.4%	442
	**Total**	**65.0%**	**2,003**

Individuals with lower educational status had higher prevalence of at least one chronic condition and more than three quarters (76.5%) of the unemployed individuals had at least one chronic condition. Study subjects with tertiary education and those with full-time employment had the lowest prevalence of at least one chronic condition as compared to other categories in the respective variables. The prevalence of at least one chronic condition was highest among study participants in the poorest wealth quintile compared to other categories in the same variable.

### Prevalence of a single chronic condition

**Figure 1:**
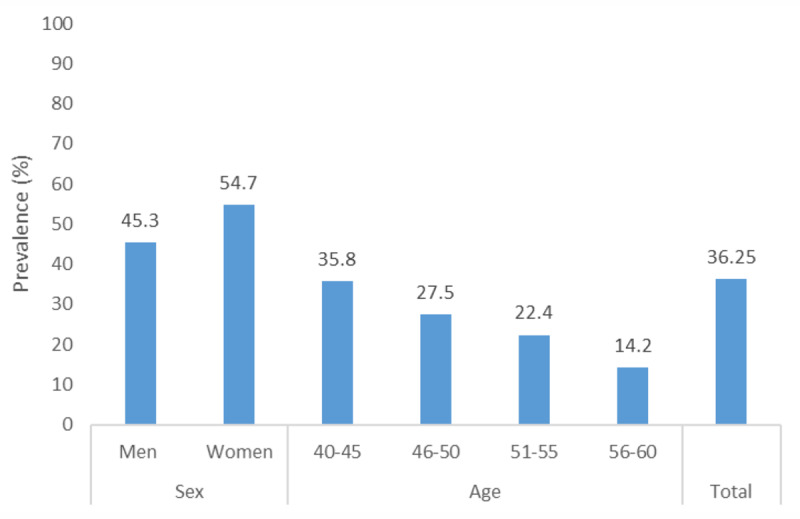
Prevalence of single chronic condition by sex and age.

The prevalence of having a single chronic condition in the study population was 36.2% (*Supplementary Appendix – Figure 1*). Out of the total 2,081 chronic conditions identified in this analysis, 726 (36.2%) of them occurred in isolation. Women had higher levels of single morbidity compared to men (54.7% vs. 45.3%). Those aged 40–45 year also had the highest level of single morbidity. Of the 726 identified single chronic conditions, the top five most prevalent were obesity (25.2%), hypertension (24.2%), drug use (12.0%), HIV (12.3%) and alcohol disorder (9.9%). Single chronic conditions from cancers and high cholesterol were not identified.

### Prevalence of multimorbidity

**Figure 2:**
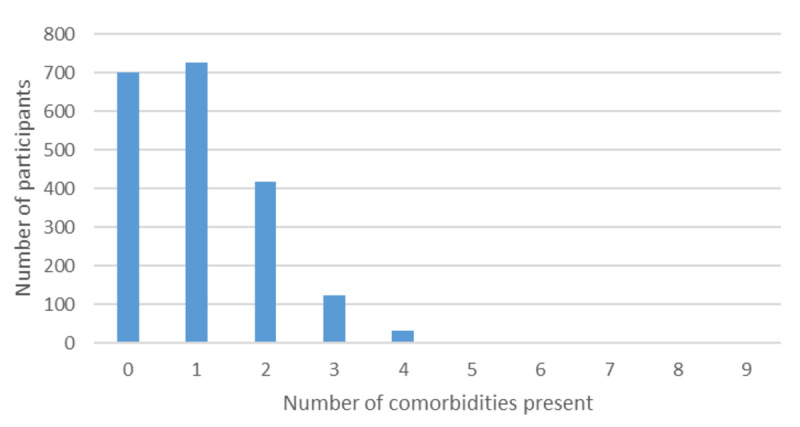
Prevalence of co-exisiting conditions.

A total of 576 study participants had two or more of the 16 chronic conditions included in this study, working out to a lifetime multimorbidity of 28.7%. The prevalence of dyads and triads of simultaneous chronic conditions was 20.8% and 6.1%, respectively. Only 1.8% of the study participants had four or more chronic condition combinations. Nine conditions were the maximum number of chronic condition combinations and this was observed in one individual (0.05%) (*Supplementary Appendix – Figure 2*). A total of 1,355 (65.1%) instances of chronic conditions were reported/identified in this multimorbidity. The three commonest chronic conditions involved in the multimorbidity complex were hypertension (20.8%), obesity (15.9%), and drug use (12.5%). The mean and median number of morbidities was 1.04 and 1.00 respectively. The chronic conditions involved in single and multimorbidity are shown in Table 4.

**Table 4:** Chronic conditions involved in instances of single- and multi-morbidity.

Single morbidity			Multimorbidity		

Chronic conditions	#	%	Chronic conditions	#	%

Obesity	183	25.2	Hypertension	282	20.8
Hypertension	176	24.2	Obesity	215	15.9
Drug use	87	12.0	Drug use	170	12.5
HIV	89	12.3	TB	157	11.6
Alcohol disorder	72	9.9	HIV	154	11.4
TB	62	8.5	Alcohol disorder	147	10.8
Asthma	18	2.5	Diabetes	53	3.9
Angina	9	1.2	Asthma	45	3.3
Diabetes	9	1.2	Angina	41	3.0
Kidney disease	10	1.4	Kidney disease	27	2.0
Stroke	5	0.7	Cancers	14	1.0
Thyroid	3	0.4	Stroke	12	0.9
Heart Failure	2	0.3	Heart attack	10	0.7
Heart attack	1	0.1	Thyroid	10	0.7
Cholesterol	–		Heart Failure	9	0.7
Cancers	–		Cholesterol	9	0.7
**Total**	**726**	**100.0**	**Total**	**1,355**	**100.0**

### Differentials of multimorbidity

Women had higher levels of lifetime multimorbidity as compared to men (31.5% vs 25.4%). Those aged greater than 50 years old had higher prevalence as compared to those 40–50 years old (32.6% vs 26.5%). Participants with tertiary level of education and those in full-time employment experienced lower prevalence of multimorbidity. Participants in the poorest wealth category had the highest level of multimorbidity compared to the other wealth quintile levels. Details of differentials of multimorbidity are displayed in Table 5.

**Table 5:** Prevalence of chronic conditions by background characteristics.

	Categories	No condition %	Single condition %	Multimorbidity %	Total (N)

Sex	Men	38.9	35.7	25.4	922
	Women	31.4	36.7	31.6	1,081
Age	≤50 years	36.8	36.7	26.5	1,254
	>50 years	31.9	35.5	32.6	749
Education	No formal education	33.1	38.3	28.6	154
	Primary	32.8	36.7	30.4	1,151
	Secondary	38.7	34.8	26.5	672
	Tertiary	46.1	38.5	15.4	26
Employment	Self-employed	32.6	38.2	29.3	946
	Full-time	47.2	33.7	19.1	267
	Part-time	33.3	40.0	26.7	45
	Informal	35.8	33.9	30.3	623
	Unemployed	23.5	38.7	37.8	119
Wealth status	Poorest	29.9	39.0	31.1	241
	Second	37.9	32.4	29.7	451
	Third	35.6	37.7	26.7	464
	Fourth	31.4	38.5	30.1	405
	Richest	37.6	35.1	28.8	442

Table 6 shows multivariate analyses results of ‘single morbidity’ against ‘no morbidity’ as the base outcome (Model 1) while Model 2 shows the results of comparing the odds of multimorbidity with that of single morbidity as the base outcome. From the results, women had significantly higher odds of having a single morbidity (OR 1.40; p value < 0.01) and multimorbidity (OR 2.46; p value < 0.001) compared to males while controlling for all other variables. Education level, wealth status, unhealthy diet and work involving sitting were neither associated with single morbidity nor multimorbidity. While holding all else constant, the odds of having a single morbidity and multimorbidity increased among those greater than 50 years of age although only significant results were observed for those with multimorbidity (OR 1.54; p value < 0.01) compared to those aged less than 50 years. Individuals in full-time employment compared to those who were self-employed were less likely to have single morbidity (OR 0.68; p value < 0.05) or multimorbidity (OR 0.62; p value < 0.05). Individuals who are unemployed had higher odds of having multimorbidity (OR 1.87; p value < 0.05) compared to those in self-employment but there were no significant differences for those experiencing single morbidity. Current use of alcohol was significantly associated with having single morbidity (OR 2.34; p value < 0.001) and much higher odds of multimorbidity (OR 5.36; p value < 0.001) compared to those not currently drinking alcohol. Similarly, current smokers had significantly higher odds of experiencing both single morbidity (OR 1.48; p value = 0.055) and multimorbidity (OR 1.95; p value < 0.05).

**Table 6:** Logistic regression outputs for differentials of multimorbidity.

Differentials	Model 1: No condition Vs single condition			Model 2: No condition Vs multimorbidity		

	Odds Ratio	[95% Conf. Interval]		Odds Ratio	[95% Conf. Interval]	

Sex (Ref: Women)	**1.40**	1.09	1.8	**2.46**	1.84	3.29
Age (Ref: 40–50 years)	1.13	0.90	1.42	**1.54**	1.2	1.97
Education (Ref: No formal education)						
Primary	0.97	0.63	1.50	1.19	0.74	1.91
Secondary	0.83	0.52	1.31	0.99	0.6	1.64
Tertiary	0.89	0.33	2.39	0.69	0.19	2.54
Ethnicity (Ref: Kamba)						
Kikuyu	1.16	0.85	1.57	**1.44**	1.03	2.02
Luhya	1.17	0.82	1.66	0.86	0.57	1.30
Luo	0.94	0.66	1.33	1.10	0.75	1.62
Others	0.68	0.45	1.05	0.74	0.46	1.19
Employment (Ref: self-employed)						
Full-time	**0.68**	0.48	0.96	**0.62**	0.41	0.94
Part-time	1.07	0.52	2.21	1.07	0.47	2.43
Informal	**0.77**	0.59	1.00	0.92	0.68	1.23
Unemployed	1.34	0.81	2.23	**1.87**	1.11	3.16
Wealth status (Ref: poorest)						
Second	0.70	0.48	1.04	0.91	0.60	1.39
Third	0.9	0.61	1.33	0.99	0.65	1.52
Fourth	1.07	0.72	1.61	1.35	0.86	2.10
Richest	0.84	0.56	1.27	1.15	0.73	1.81
Behavioural risk factors						
Current use of alcohol (Ref: No)	**2.34**	1.65	3.32	**5.36**	3.72	7.70
Current smoking (Ref: No)	1.48	0.99	2.22	**1.95**	1.28	2.97
Unhealthy diet (Ref: No)	0.93	0.73	1.19	0.96	0.73	1.25
Work involves sitting (Ref: No)	1.08	0.87	1.35	0.93	0.74	1.19

## Discussion

To our knowledge, this is the first study in the country specific context to estimate multimorbidity and its associations at a population level and within a HDSS providing potential for wave data points for temporal association studies between socio-demographic factors and the occurrence of chronic conditions. The results show that multimorbidity is prevalent (28.7%) in the urban slum population of Kenya with 65% of the study population having experienced at least one chronic condition. The findings suggest that several factors including age, gender, ethnicity, employment, individuals consuming alcohol and those smoking are associated with both single and multimorbidity. These findings contribute to the evidence base from sub-Saharan Africa on the prevalence and determinants of multimorbidity. The findings will also inform the design of integrated chronic care disease models for management of non-communicable diseases as well as communicable/non-communicable disease dichotomy. In most LMICs, resources are invested in running vertical programs for TB and HIV control and management but, the results provided here demonstrate that many patients present with more than one chronic disease condition; NCDS in most cases. Sustainable Development Goal 3 aims to ‘ensure healthy lives and promote well-being for all at all ages’ by 2030. However, this may not be achievable if governments do not prioritize and plan for the health needs of their citizens.

Among the 2,003 respondents included in the analyses, 2,081 episodes of having had a chronic condition at least once in their lifetime were identified. The high prevalence (28.7%) of multimorbidity in this study is very concerning particularly because it is among an economically disadvantaged population [22,23]. The prevalence found in the current study is higher than what has been reported in high-income countries [6,24], and in South Africa (22.5%) [25], but it was lower than what was found in a study conducted in Ghana (38.8%) [9]. In the current study, the most prevalent chronic conditions were hypertension (23%). This is similar to what was found in South Africa and Ghana [9,25].

There is increasing awareness on the fact that the world’s population is rapidly ageing. Even though much focus on health systems and wellbeing policies for the aging population in developed nations is substantially advanced, little consideration has been given to issues of old age health in sub-Saharan Africa. Despite being the youngest region, SSA’s demographic transition is real with pointers of unprepared emerging health trends highlighted by multimorbidity among others posing a greater economic burden to the region and countries’ health system framework and economic wellbeing [21,22,23]. From the study findings, a higher proportion of women than men, older individuals, respondents with lower levels of education, unemployed respondents and respondents in the poorest wealth category had a higher prevalence of at least one chronic condition compared to their peers. Equally, the occurrence of multimorbidity or having had at least two or more chronic conditions was higher among women, older individuals, participants with no formal and primary level of education (compared to those with secondary and tertiary), unemployed respondents and individuals in the lowest wealth category. In the multimorbidity complex, hypertension, obesity, and drug use were among the highest chronic conditions identified. These determinants are consistent across a diverse range of other studies where multimorbidity is reported more among women and a lower burden of multimorbidity among those with higher education [5,9,26].

The results further suggest that several factors including older age, being female, unemployment, current alcohol consumers and current smokers were associated with multimorbidity. Older adults (50+ years) had significantly higher odds of experiencing a multimorbidity compared to those aged 40–50 years. The high prevalence of multimorbidity in older people has also been reported in other settings and populations [4,9,25]. This is somewhat expected as those that live longer have more exposure to chronic condition risk. Women had higher odds of multimorbidity compared to men in this study. This finding is similar to findings from other settings [4,9,27]. Likely reasons for this observation maybe due to women’s physiologic makeup and the changes that occur during women’s reproductive years that may predispose them more to some of the conditions reported in this study such as overweight and obesity [28]. A study in the same setting also reported that women engaged more in sedentary activities and had higher levels of general obesity and normal-weight central obesity compared to men, which may predispose them to other chronic conditions [29,30]. Other reasons include women’s greater use of health facilities thus providing an opportunity for an increased chance for diagnosis of illness [31,32].

Unemployment in this study was associated with multimorbidity. The reason behind this finding is not clear, but it could possibly mean that those who are unemployed are too sick to hold employment. This finding will need to be investigated further. Multimorbidity is reported to be positively associated with country GDP per capita suggesting that people have more freedom to make better lifestyle choices and better social conditions as well as ways of communities/ethnicities socialization among others [5,33]. Hence, probable pointers to the observed odds. Such health and social inclined outcomes have been shown to have the potential to markedly influence or be influenced by exposure to risk factors, such as tobacco smoking and alcohol consumption [33,34,35].

The strength of this study is that it is the first study in an urban slum population in Kenya to estimate multimorbidity and its determinants. Key limitations of these analyses include data were collected through a self-report and there was no information on the exact time when the chronic conditions occurred. Relying on self-reported health in communities with low health literacy could potentially introduce a wide margin of error. The measure of multimorbidity in this study was a mere count that did not consider the intensity or severity of disease. Having more than one disease may not necessarily mean a worse health outcome if the conditions are under control. Further, the data for this study was from individuals aged between 40 and 60 years, living in two urban slums thus limiting the extent to which the results of the present study can be generalized to the informal settlement population. Therefore, the findings of this study should be interpreted with the above limitations in mind. Nonetheless, valuable information on the prevalence and factors associated with multimorbidity was obtained and this makes a significant contribution to the body of knowledge in this area to inform the design of interventions for integrated disease management programs.

### Conclusions and recommendations

Multimorbidity is prevalent in informal settlements with about one in every three people aged 40–60 years having more than two conditions while about a one third of the study population had none of the 16 chronic conditions under consideration. Hypertension, obesity, drug use, HIV, TB, and alcohol disorder were the most prevalent chronic conditions, often occurring concurrently. These chronic conditions closely match the risk factors identified as major contributors to disability adjusted life years (DALYs), some of which develop from as early as 10 years of age as reported by Gore and colleagues [36]. High blood pressure, overweight/obesity, alcohol use are the 1^st^, 5^th^, and 8^th^ leading causes of deaths globally [37].

Multimorbidity is a public health concern due to its association with high costs to healthcare systems and individuals, strain on quality of health care and increased risk of mortality, disability, or poor quality of life. Consequently, this growing threat requires prioritization by states and other key actors, considering the broader implications of multimorbidity, such that resources and the public health system are well placed to adequately address this issue. Concerted efforts are needed to develop strategies for the planning, prevention and management of modifiable risk factors that drive the high prevalence of multimorbidity. More efforts should also be placed in designing integrated chronic care models. Further research is also necessary in different types of settings as well as follow-up surveys to monitor trends.
